# Association Between Dietary Patterns and Frailty in Chinese Longevity Regions: Evidence From the HABCS

**DOI:** 10.1093/geroni/igaf122.3711

**Published:** 2025-12-31

**Authors:** Shi Yin, Wuqi Wang, Yuebin Lv, Xiaoming Shi

**Affiliations:** The First Affiliated Hospital of USTC, University of Science and Technology of China, Hefei, Anhui, China (People’s Republic); School of Public Health, Anhui Medical University, Hefei, Anhui, China (People’s Republic); Chinese Center for Disease Control and Prevention, Beijing, Beijing, China (People’s Republic); Chinese Center for Disease Control and Prevention, Beijing, Beijing, China (People’s Republic)

## Abstract

Frailty, a geriatric syndrome characterized by reduced physiological reserve and increased vulnerability to stressors, has been associated with dietary habits, though previous studies have focused mainly on Western nations and select Asian regions, leaving a significant gap in systematically examining the distinct dietary patterns and their associations with frailty in Chinese longevity regions. This study leveraged data from 3242 older adults in the Chinese Healthy Ageing and Biomarkers Cohort Study (HABCS) to explore dietary patterns and associations with frailty. Dietary intake was assessed via a validated Food Frequency Questionnaire (FFQ), and frailty was defined using a 39-item index (cutoff >0.25). Mediation analysis examined serum biomarkers (superoxide dismutase [SOD], high-sensitivity C-reactive protein [hsCRP]). Principal Component Analysis revealed four major dietary patterns among older adults in Chinese longevity regions, which were Pattern 1, Wheat-fruits and Vegetables-Dairy and Eggs (15.9% variance), Pattern 2, White Rice-Aquatic products-Red meat and Vegetables (13.3% variance), Pattern 3, Pickled Foods (8.4% variance), and Pattern 4, Grains-Starchy vegetables-Legumes-White Meat (10.0% variance), collectively explaining 47.5% of variance. Pattern 2 was protective against frailty (OR = 0.56; 95%CI: 0.45–0.72), while Pattern 4 increased frailty risk (OR = 1.43; 95%CI: 1.32–1.80). Patterns 1 and 3 showed no significant associations. Mediation analysis revealed that hsCRP mediated 2.7% (P = 0.028) of Pattern 2’s protective effect; SOD had no significant role. The findings suggest that a diet rich in rice, aquatic products, red meat, and vegetables may reduce frailty risk, potentially due to high-quality protein and anti-inflammatory components. This highlights the importance of region-specific dietary guidance for healthy aging.

